# Epidemiological investigation into the prevalence of abnormal inter-arm blood pressure differences among different ethnicities in Xinjiang, China

**DOI:** 10.1371/journal.pone.0188546

**Published:** 2018-01-18

**Authors:** Ling Sun, Ting Zou, Bao-Zhu Wang, Fen Liu, Qing-Hua Yuan, Yi-Tong Ma, Xiang Ma

**Affiliations:** 1 Department of Cardiology, First Affiliated Hospital of Xingjiang Medical University, Urumqi, China; 2 Department of Cardiology, Fifth Affiliated Hospital of Xinjiang Medical University, Department of Cardiology, Henan, Urumqi, China; 3 Xin Jiang Key Laboratory of Cardiovascular Disease, Clinical Medical Research Institute of the First Affiliated Hospital of Xinjiang Medical University, Urumqi, China; Baker IDI Heart and Diabetes Institute, AUSTRALIA

## Abstract

**Objectives:**

The prevalence of and risk factors for IAD among different ethnicity groups was unknown. Our aim was to investigate the prevalence of and risk factors for IAD among Han, Uygur and Kazakh ethnicities in Xinjiang. China.

**Methods:**

In total, 14,618 adult participants (7,799 males and 6,819 females) were recruited from the Cardiovascular Risk Survey. A 4-stage stratified cluster random sampling method was used. The participants’ personal information and medical history were assessed by questionnaire. IAD was diagnosed by a noninvasive arteriosclerosis analyzer.

**Results:**

The prevalence of abnormal IAD among the general population was 14.3%, with 12.5% in the Han, 14.9% in the Uygur, and 16.4% in the Kazakh populations. The prevalence of abnormal IAD among the hypertensive population was 19.4%, with 17.0% in the Han, 18.1% in the Uygur, and 22.7% in the Kazakh populations. The prevalence of abnormal IAD increased with age (all P < 0.01) but was not significantly different between the genders (all P> 0.05). Multivariate logistic regression analysis showed that age more than 45 years, obesity and hypertriglyceridemia were significantly associated with a higher prevalence of IAD. There were different risk factors for abnormal IAD in different ethnicities. Middle or old age, obesity, ABI and diabetes mellitus were risk factors for the Han population, smoking was a risk factor in the Uygur population, and obesity and PAD were risk factors in the Kazakh population.

**Conclusion:**

The prevalence of abnormal IAD in the Kazakh participants was higher than that in the Han and Uygur populations among both the general population and the hypertensive population in Xinjiang, China. The main risk factors of IAD were age, obesity, and triglyceride levels. Different ethnicities had different kinds of risk factors for IAD.

## Introduction

Recent studies have shown that the inter-arm blood pressure difference (IAD) is associated with not only peripheral artery disease but also subclavian artery stenosis, cardiovascular morbidity and mortality, and all-cause mortality [1–3].

IAD is defined as the absolute difference in averaged BPs between the left and right arms, and an IAD≥10 mm Hg is considered to be significant [4,5]. Significantly increased IAD increases the risk for cardiovascular disease (CVD) [6,7]. Importantly, IAD is a more easily obtained, economical and non-invasive parameter than traditional risk factors [4,8].

The Hypertension Genetic Epidemiology Network study also found [9] that compared with the general population (9.2%), people with hypertension have a higher prevalence of IAD (14.2%). The Kimura A’ study noted that IAD is associated with risk factors for arteriosclerosis such as hypertension, hypercholesterolemia, obesity and metabolic abnormalities [10]. However, there is a lack of epidemiological data assessing the prevalence of IAD in populations with different ethnic backgrounds. Therefore, the purpose of this study was to estimate the abnormal prevalence of IAD and to analyze its risk factors among the Han, Uygur, and Kazakh populations in Xinjiang, China.

## Methods

### Ethics statement

This study was approved by the Ethical Committee of the First Affiliated Hospital of Xinjiang Medical University and was carried out according to the Declaration of Helsinki. Each participant signed a written informed consent form.

### Participants

All the participants were selected from the Cardiovascular Risk Survey (CRS) conducted during October 2007 and March 2010. The study population and methods have been described in detail in a previous study [11–13]. Briefly, the CRS was a multiple ethnicity, community-based, cross-sectional study. We use 4-stage stratified cluster random sampling to select a representative sample of the general population of Chinese Hans, Uygurs, and Kazakhs of this area. Seven cities (Urumqi, Kelamayi, Hetian, Zhaosu, Fukang, Tulufan, and Fuhai) were chosen and, based on the government record of registered residence, one participant was randomly selected from each household. In this investigation, out of the total, 14618 participants (5757 Han, 4767 Uygur, and 4094 Kazakh Chinese), were randomly selected from 26 villages of those seven cities and invited to participate. Those whose data were incomplete were excluded. Finally, 11,239 subjects (5145 Hans, 2456 Uygurs, and 3638 Kazakhs) completed the survey and examination.

### Survey contents

The investigations were carried out with the same survey program after the same training. The survey included two parts, the completion of the questionnaire form and a physical examination, which were conducted by standardized cardiovascular specialists. The questionnaire mainly included questions about the subject’s general condition, occupation, labor intensity, personal history of hypertension, family history and so on. The physical examination section included measurements of blood pressure, heart rate, electrocardiogram, height, weight, waist circumference, abdominal circumference, and hip circumference.

### Measurement methods

Height was measured using a centimeter ruler. The ruler was placed perpendicular to the ground and affixed to the wall. The subjects were instructed to keep their line of sight forward. The examiner measured and recorded the number level with the top of the subject’s head using the right-angle side of a triangular ruler. For the weight measurement, the scale was zeroed before each use. Subjects fasted and urinated before the measurement. For the blood pressure (BP) measurement, the subjects stopped smoking 15 min before the test and spent at least 5 minutes resting in the sitting position. The BP measurement was performed three times using the right arm, and the mean BP value of the three readings was used for statistical analysis. For the measurements of IAD and the ankle-brachial index (ABI), the subjects were supine, their limbs were wrapped with a special double-cuff. Then, the blood pressure in the limbs was measured in a synchronized way by a Japanese Omron-Colin noninvasive arteriosclerosis analyzer VP-1000 (BP-20w3RPE II), and the IAD was calculated by the subtracting the blood pressure value of the left upper limb from that of the right upper limb. The ABI value was automatically derived by dividing the ankle blood pressure by the higher value of blood pressure in the right or left upper arm.

### Personnel training and quality control

All survey forms were reviewed daily and maintained by a specially assigned person. Data were input by various researchers after a unified standardized training. The database was established, and the statistical analysis was conducted. Investigators were supervised during the investigation. The data was recorded twice with the unified database software. Each 100 questionnaire forms were used as a batch of materials for comparison. Modification was performed according to the original table in the case of any inconsistency. The process was repeated until the data were correct.

### Laboratory testing

Study subjects fasted for 12 h, and 5 mL of venous blood was collected the morning of the day of the physical examination. Blood tests included total cholesterol (TC), triglyceride (TG), fasting blood glucose (FBG), high-density lipoprotein cholesterol (HDL-c), low-density lipoprotein cholesterol (LDL-c), blood urea nitrogen (BUN), creatinine (Cr), and uric acid (UA). The mentioned inspection and testing were performed by well-trained professionals.

### Definition of IAD

IAD was defined as the absolute difference in averaged BPs between the left and right arm, and an IAD ≥ 10 mm Hg was significant [5].

### Definition of hypertension

According to the criteria in the China hypertension guidelines (2010) [14], the definition of hypertension was as follows: systolic blood pressure (SBP) ≥ 140 mm Hg (1 mm Hg = 0.133 kPa) and (or) diastolic blood pressure (DBP) ≥ 90 mm Hg. Alternatively, patients were measured and had normal blood pressure after nearly 2 weeks of taking antihypertensive drugs, excluding secondary hypertension.

### Definition of ankle brachial index

The ankle-brachial index (ABI) was defined the ankle blood pressure divided by the higher value of blood pressure in the right or left upper arm. The ABI was classified as ABI ≤ 0.90 or ≥ 1.40, with a lower ABI as the patient's ABI [15,16].

### Definition of diabetes mellitus

According to the World Health Organization (WHO) Diabetes Classification and Guidelines [17], diabetes was defined as FBG ≥ 7.0 mmol/L or a history of diagnosed diabetes.

### Definition of dyslipidemia

Dyslipidemia was determined by a self-report of using anti-hyperlipidemic medications or by having one of the following four lipid abnormalities: TC > 6.22 mmol/L (240 mg/dL) was defined as hypercholesterolemia, TG > 2.26 mmol/L (200 mg/dL) was defined as hypertriglyceridemia, LDL-c > 4.14 mmol/L (160 mg/dL) was defined as a high concentration of LDL-c, and HDL-c < 1.04 mmol/L (40 mg/dL) was defined as a low concentration of HDL-c [18].

### Definition of obesity

The body mass index is calculated as body weight (kg)/height^2^ (m^2^). Patients with 18.5 kg/m^2^ ≤ BMI < 24 kg/m^2^ were normal weight, patients with 24 kg/m^2^ ≤ BMI < 28 kg/m^2^ were overweight, and patients with BMI ≥ 28 kg/m^2^ were obese [19].

### Definition of smoking and drinking

Participants who reported regular smoking in the previous 6 months were considered current smokers, and those who reported regular drinking in the last 6 months were considered alcohol users [20].

### Statistical analysis

The data were verified and corrected by two staff members using EpiData 3.02 software (EpiData Association, Odense, Denmark). The statistical analysis was conducted using Social Sciences SPSS for Windows version 22. (SPSS, Inc., IL, USA). Continuous variables were expressed as the means ± standard deviation, numerical data were expressed as rates, and a chi-square test (χ^2^) was used to evaluate differences between groups. Age standardization was performed according to the census of the Xinjiang Uygur autonomous region in 2010. The risk factors for IAD were analyzed using a multivariate unconditional logistic regression, and the significance level alpha value was set to 0.05.

## Results

### General characteristics of the included population

The survey included a total of 11,239 people [5294 (47.7%) males and 5945 (52.9%) females]. The average age of all subjects was 50.60 ± 12.50 years. A total of 5145 cases (45.8%), 2495 cases (21.9%) and 3638 cases (32.4%) were from Han, Uygur, and Kazakh populations, respectively. The cases were divided into five groups according to the age composition of the total population. Patients with complete information were included in the final analysis, and their general information is presented in Table 1. Gender, blood pressure, BMI, TGs, TC and other indicators were significantly different among different ethnic groups. The distribution of epidemiological data of the IAD normal and abnormal groups is shown in Table 2.

**Table 1:** General characteristics of study participants.

Group	Han (n = 5145)	Uygur (n = 2456)	Kazakh (n = 3638)
Age, years	52.29±12.62	52.26±12.18*	48.56±11.81*☨
Height, cm^2^	163.14±8.54	161.65±8.27	162.57±8.74*
BMI, kg/m^2^	25.14±3.51	26.29±4.31*	26.49±4.76*☨
SBP, mm Hg	132±20	136±21*	140±25*
DBP, mm Hg	85±16	84±16*	88±20☨
ABI	1.07±0.09	1.03±0.11*	1.05±0.10*☨
PWV	1521.82±400.68	1518.29±376.35	1553.26±397.74*☨
FBG, mmol/L	5.33±1.78	5.12±1.51*	5.17±1.71*☨
Smoking	1611(31.3%)	652(26.6%)	1302(35.9%)
Drinking	986(19.2%)	388(15.8%)	517(14.2%)
TG, mmol/L	1.72±1.45	1.68±1.18	1.21±0.89*☨
HDL-c, mmol/L	1.25±0.46	1.26±0.49	1.29±0.42*
LDL-c, mmol/L	2.86±0.90	2.90±0.94	2.90±0.93
TC, mmol/L	4.68±1.08	4.60±1.24	4.76±1.16
UA, μmol/L	306.45±87.52	258.49±79.71*	260.71±79.40*☨
Cr, μmol/L	75.79±26.91	73.93±34.01*	70.68±19.29*☨

BMI, body mass index; SBP, systolic blood pressure; DBP, diastolic blood pressure; ABI, ankle-brachial index; PWV, pulse wave velocity; FBG, fasting blood glucose; HDL-c, high-density lipoprotein cholesterol; LDL-c, low-density lipoprotein cholesterol; TC, total cholesterol; TG, triglycerides; Cr, creatinine; UA, uric acid.

*p<0.05 versus the Han participants

☨p<0.05 versus the Uygur participants.

**Table 2:** Distribution of epidemiological data of IAD normal and abnormal groups.

Groups	IAD < 10 mm Hg	IAD ≥ 10 mm Hg	t / χ^2^	P
Age, years	50.67±12.34	53.52±12.37	-8.56	0.79
Height, cm^2^	162.71±8.53	162.18±8.78	2.27	0.15
Weight, kg	68.17±13.19	70.70±14.10	-6.68	<0.01
BMI, kg/m^2^	25.66±4.11	26.79±4.43	-9.45	<0.01
SBP, mm Hg	134±21	148±27	-19.68	<0.01
DBP, mm Hg	85±17	92±19	-13.78	<0.01
ABI	1.05±0.10	1.05±0.11	2.37	<0.01
PWV	1746.31±429.94	1851.47±437.37	4.21	0.01
FBG, mmol/L	5.14±1.59	5.33±2.30	-3.13	<0.01
Smoking	3077(32.0%)	488(30.5%)	1.40	0.24
Drinking	1627(16.9%)	264(16.5%)	0.17	0.68
TG, mmol/L	1.53±1.25	1.63±1.30	-2.78	0.07
TC, mmol/L	4.68±1.13	4.74±1.20	-1.95	0.03
HDL-c, mmol/L	1.27±0.45	1.25±0.48	0.21	0.82
LDL-c, mmol/L	2.88±0.92	2.88±0.93	-0.18	0.32
Cr, μmol/L	73.83±27.05	73.21±25.21	0.84	0.78
UA, μmol/L	280.87±86.46	283.44±86.57	-1.08	0.53

BMI, body mass index; SBP, systolic blood pressure; DBP, diastolic blood pressure; ABI, ankle-brachial index; PWV, pulse wave velocity; FBG, fasting blood glucose; HDL-c, high-density lipoprotein cholesterol; LDL-c, low-density lipoprotein cholesterol; TC, total cholesterol; TG, triglycerides; Cr, creatinine; UA, uric acid.

### Analysis of the prevalence of IAD in different sex and age groups

The total detection rate of interarm pressure differences was 14.3%. The detection rate of IAD was 14.5% in males and 14.0% in females. The prevalence of IAD was not significantly different between males and females (χ^2^ = 0.57, P = 0.45). A significant difference was observed in the prevalence of IAD among different age groups (χ^2^ = 75.45, P < 0.01). The prevalence of IAD in the age groups of 35–44, 45–54, 55–64, 65–74, and over 75 years was 10.8%, 14.9%, 16.3%, 18% and 18.5%, respectively (Table 3).

**Table 3:** Comparison of abnormal IAD prevalence rates of the general population among three ethnic groups (n, %).

Age/years	Han (n = 642)		Total	Uygur (n = 366)		Total	Kazakh (n = 595)		Total
	Male	Female		Male	Female		Male	Female	
35–44 y	94	72	166	34	50	84	94	95	189
	(10.3%)	(8.0%)	(9.2%)	(13.0%)	(11.7%)	(12.2%)	(13.0%)	(11.3%)	(12.1%)
45–54 y	73	83	156	59	59	118	82	91	173
	(12.1%)	(12.6%)	(12.4%)	(18.0%)	(13.9%)	(15.7%)	(17.8%)	(17.4%)	17.6%
55–64 y	47	88	135	36	54	90	63	73	136
	(11.9%)	(15.4%)	(13.9%)	(12.9%)	(8.1%)	(15.5%)	(20.5%)	(20.6%)	(16.3%)
65–74 y	76	72	148	32	28	60	43	34	77
	(18.8%)	(13.0%)	(16.7%)	(16.8%)	(17.8%)	(17.3%)	(22.1%)	(21.5%)	(21.8%)
more than 75y	19	18	37	6	8	14	11	9	20
	(14.4%)	(20.7%)	(16.9%)	(11.1%)	(22.9%)	(15.7%)	(23.9%)	(31.0%)	(26.7%)
Total	309	333	642	167	199	366	293	302	595
	(12.6%)	(12.3%)	(12.5%)	(15.0%)	(14.8%)	(14.9%)	(16.9%)	(15.9%)	(16.4%)
Standard prevalence	309	333	642	167	199	366	293	302	595
	(11.9%)	(11.6%)	(11.8%)	(14.6%)	(14.3%)	(14.3%)	(16.7%)	(16.2%)	(15.7%)

The incidence of abnormal IAD was higher for the Kazakh ethnic group than for the Uygur and Han ethnic groups; there were differences between the three groups (*χ*^2^ = 70.35, *P* < 0.01). A significant difference was observed in the prevalence of IAD among different age groups (*χ*^2^ = 72.45, *P* < 0.01).

### Analysis of the prevalence of IAD among different ethnicities

The prevalence of IAD was 12.5% in the Han subjects, 14.9% in the Uygur subjects, and 16.4% in the Kazakh subjects, respectively. After age standardization, the prevalence of IAD abnormality was 11.8%, 14.3%, and 16.4%, respectively, in the three populations (Table 3). The prevalence of IAD abnormalities among the three ethnic groups was significantly different (χ^2^ = 70.35, P < 0.01).

### Analysis of the prevalence of IAD in the hypertensive population of different ethnicities

The total prevalence of IAD was 19.4% in patients with hypertension. The prevalence of IAD in hypertensive Han, Uygur and Kazakh populations were 17.0%, 18.1% and 22.7%, respectively. After age standardization, the prevalence of IAD abnormality was 15.5%, 18.6%, and 21.4%, respectively, in the three groups. The prevalence of IAD was significantly different among those three ethnic groups (χ^2^ = 70.35, P < 0.01). The prevalence of IAD in hypertensive males was 19.0%, while it was 19.7% in hypertensive females, and there was no significant difference between the prevalence according to gender (χ^2^ = 0.34, P = 0.56). The prevalence of IAD among different age groups in the hypertensive population was significantly different (χ^2^ = 10.76, P = 0.03) (Table 4). The prevalence of IAD was 15.7%, 19.8%, 20.3%, 20.6%, and 21.6% in the 35–44, 45–54, 55–64, 65–74, and over 75 years age groups, respectively.

**Table 4:** Comparison of the prevalence of abnormal IAD in the hypertensive population among the three ethnic groups (n, %).

Age/years	Han (n = 328)		Total	Uygur (n = 188)		Total	Kazakh (n = 394)		Total
	Male	Female		Male	Female		Male	Female	
35–44 y	32	19	51	8	16	24	48	28	76
	(12.7%)	(15.0%)	(13.5%)	(13.6%)	(18.2%)	(16.3%)	(18.9%)	(15.6%)	(17.6%)
45–54 y	35	27	62	24	36	60	59	62	121
	(15.4%)	(16.1%)	(15.7%)	(21.1%)	(18.5%)	(19.4%)	(23.0%)	(22.4%)	(23.0%)
55–64 y	26	59	85	22	29	51	50	63	66
	(15.2%)	(19.6)	(18.0%)	(15.8%)	(17.5%)	(16.7%)	(23.80%)	(26.4%)	(24.9%)
65–74 y	48	55	103	20	22	42	38	28	26
	(1.7%)	(17.0%)	(18.9%)	(18.2%)	(21.40%)	(19.7%)	(26.4%)	(23.0%)	(24.8%)
more than 75y	13	14	27	5	6	11	10	8	18
	(16.7%)	(23.7)	(19.7%)	(13.2%)	(25.0%)	(17.7%)	(27.0%)	(34.8%)	(30.0%)
Total	154	174	328	79	109	188	205	189	394
	(16.2%)	(17.8%)	(17.0%)	(17.2%)	(18.9%)	(18.1%)	(23.0%)	(22.50%)	(22.7%)
Standard prevalence	154	174	328	79	109	188	205	189	394
	(14.4%)	(16.5%)	(15.5%)	(16.4%)	(18.7%)	(18.6%)	(22.0%)	(20.7%)	(21.4%)

Differences were observed among the three groups in the hypertensive population (*χ*^2^ = 70.35, P < 0.01). A significant difference was observed in the prevalence of IAD among different age groups (*χ*^2^ = 10.76, P = 0.03).

### Analysis of the relevant risk factors for IAD

Multivariate unconditional logistic regression analysis was used to analyze the data. The valuable and empirical variables were included in the multivariate unconditional logistic regression analysis (Table 5), and the variables were introduced in the equation. Age, ethnicity, obesity, and ABI were the risk factors for IAD after adjusting for factors such as investigation site, sex, marital status, and diet. Compared with the group of subjects aged 35–44 years (OR = 1), the risk of developing IAD increased by 1.71-fold in the groups of subjects with ages greater than 75 years (OR = 1.71, 95% CI: 1.25–2.33). In the obese group, the risk of IAD was 1.41-fold higher (OR = 1.41, 95% CI: 1.21–1.65) than that of the normal population. Risk factors for IAD in the hypertensive population were consistent with those in the general population (Table 6). A multiple regression analysis was also performed for differences in the prevalence of IAD abnormalities among the three ethnic groups. No significant differences were observed in the risk factors for IAD among the different ethnic groups. Age, ethnicity, obesity, and ABI were the common risk factors for IAD among the three ethnicities.

**Table 5:** Multivariate unconditional logistic regression analysis of risk factors for abnormal IAD in the general population.

Factor	B	S.E.	wald	df	P	OR	95% CI
Sex	0.00	0.08	0.00	1	0.10	1.00	0.86–1.16
Age			22.70	4	0.00		
35–44 y						1	
45–54 y	0.23	0.08	8.60	1	0.03	1.26	1.08–1.48
55–64 y	0.30	0.09	11.50	1	0.01	1.91	1.14–1.61
65–74 y	0.42	0.10	16.90	1	0.00	2.03	1.24–1.85
More than 75y	0.53	0.16	11.30	1	0.01	2.88	1.25–2.33
Ethnicity			12.20	2	0.01		
Han						1	
Uygur	0.07	0.09	0.58	1	0.45	0.67	0.90–1.28
Kazakh	0.26	0.08	11.12	1	0.01	1.00	1.12–1.51
Smoking	-0.08	0.08	0.91	1	0.34	0.89	0.80–1.08
Drinking	0.03	0.09	0.09	1	0.77	1.17	0.86–1.23
BMI			19.70	2	0.00		
Normal						1	
Overweight	0.15	0.07	3.93	1	0.47	1.16	1.00–1.34
Obesity	0.35	0.08	19.30	1	0.00	1.41	1.21–1.65
Diabetes	0.14	0.11	1.47	1	0.23	1.15	0.92–1.43
TG	0.14	0.07	4.27	1	0.04	1.15	1.01–1.32
TC	-0.06	0.07	0.79	1	0.37	0.94	0.83–1.07
HDL-c	-0.02	0.06	0.13	1	0.72	0.98	0.87–1.11
LDL-c	0.03	0.64	0.22	1	0.64	1.03	0.91–1.17
ABI	-0.83	0.36	5.35	1	0.02	0.04	0.22–0.88
Constant	-1.28	0.46	7.86	1	0.05	0.28	

BMI, body mass index; HDL-c, high-density lipoprotein cholesterol; LDL-c, low-density lipoprotein cholesterol; TC, total cholesterol; TG, triglycerides.

**Table 6:** Multivariate unconditional logistic regression analysis of risk factors for IAD in the hypertensive population.

Factor	B	S.E.	wald	df	P	OR	95% CI
Sex	0.00	0.10	0.00	1	1.00	1.00	0.83–1.21
Age			5.54	4	0.24		
35–44 y						1	
45–54 y	0.14	0.12	1.27	1	0.26	1.15	0.90–1.46
55–64 y	0.20	0.13	2.49	1	0.12	1.22	0.95–1.56
65–74 y	0.27	0.13	4.15	1	0.04	1.31	1.01–1.70
More than 75y	0.35	0.19	3.49	1	0.06	1.43	0.98–2.07
Ethnicity			12.2	2	0.01		
Han						1	
Uygur	-0.05	0.11	0.11	1	0.68	0.96	0.77–1.19
Kazakh	0.26	0.08	0.10	1	0.01	1.33	1.11–1.61
Smoking	-0.08	0.08	0.11	1	0.49	0.93	0.76–1.14
Drinking	0.03	0.13	0.04	1	0.84	1.03	0.81–1.31
BMI			7.69	2	0.02		
Normal						1	
Overweight	-0.03	0.11	0.06	1	0.81	0.98	0.79–1.20
Obesity	0.21	0.11	3.91	1	0.048	1.23	1.00–1.52
Diabetes	0.06	0.14	0.20	1	0.66	1.06	0.81–1.40
TG	0.11	0.09	1.52	1	0.22	1.03	0.79–1.35
TC	-0.02	0.08	0.08	1	0.78	0.98	0.83–1.15
HDL-c	0.01	0.09	0.01	1	0.95	1.01	0.85–1.19
LDL-c	-0.03	0.08	0.08	1	0.77	0.98	0.83–1.15
ABI	-1.41	0.47	8.89	1	0.01	0.24	0.09–0.62
Constant	-0.52	0.55	0.89	1	0.35	0.60	

ABI, ankle-brachial index; BMI, body mass index; HDL-c, high-density lipoprotein cholesterol; LDL-c low-density lipoprotein cholesterol; TC, total cholesterol; TG, triglycerides.

### Comparison of IAD risk factors in the three ethnicity groups

Multivariate unconditional logistic regression analysis was used on all related risk factors to recognize any existing differences in those risk factors that might explain the difference in IAD prevalence among the three ethnicities. The comparison was made between participants with abnormal IAD values in the Han (n = 595), Uygur (n = 320) and Kazakh (n = 541) populations and their corresponding IAD-normal participants, and the results are shown in S1 Table (S1 Table). In the Han population, middle and old age (65–74 years old, OR = 1.640, 95% CI: 1.243–2.164), obesity (OR = 1.469, 95% CI: 1.146–1.884), ABI (OR = 0.229, 95% CI: 0.076–0.695) and diabetes mellitus (OR = 1.340, 95% CI: 1.007–1.783) remained risk factors. In the Uygur population, smoking (OR = 3.626, 95% CI: 2.295–5.729) remained a risk factor, and in the Kazakh population, obesity (OR = 1.490, 95% CI: 1.154–1.295) and PAD (OR = 1.603, 95% CI: 1.014–2.533) remained risk factors for IAD.

## Discussion

In the present study, we found that the abnormal prevalence of IAD was 14.3%, which was higher than the prevalence reported in previous studies (10.7% [21] and 3.7% [22]). Meanwhile, our data also illustrated that the prevalence of abnormal IAD in the hypertensive population was 19.4%, which was higher than the prevalence reported in studies by Kim SA et al. (7.7%) [5] and Clark CE et a1. (11.2%) [23]. This might be due to differences in study populations and the methods used for BP measurement.

Our study showed differences in the prevalence of abnormal IAD according to ethnicity. The prevalence of abnormal IAD in the Han, Uygur and Kazakh populations was 12.5%, 14.9% and 16.4%, respectively. The prevalence of abnormal IAD in the Kazakh population was significantly higher than that in the Han and Uygur populations. The present data also showed that the prevalence of abnormal IAD has differences in the hypertensive population according to ethnicity. Of the three ethnicities, the highest rate of abnormal IAD was 22.7% in the Kazakh ethnic group. This difference may be due to a higher prevalence of hypertension among the Kazakh population in Xinjiang [24].

Many studies have demonstrated that arterial elasticity decreases and peripheral vascular resistance increases with age, which are associated with atherosclerosis. This could account for the prevalence of abnormal IAD in the elderly population [25,26]. In the present study, we found significant differences in the prevalence of abnormal IAD according to age, with higher prevalence in older age groups. The rates of abnormal IAD in different age groups (35–44 years, 45–54 years, 55–64 years, 65–74 years and > 75 years) were 10.8%, 14.9%, 16.3%, 18% and 18.5%, respectively., People over the age of 45 are at especially high risk for abnormal IAD and particular attention should be paid to measurements of inter-arm differences in BP in these people. Furthermore, our study showed that gender was not a significant factor associated with a large IAD, which is consistent with the results of other reports [27]. It is unclear whether this was influenced by changes associated with female hormones and menopause [28,29].

In the present study, obesity and hyperlipidemia were associated with a higher risk of abnormal IAD. Obesity is an increasing epidemic in both adults and children, and these obese individuals often have concomitant hypertension. Obesity is associated with increased blood viscosity, which increases the rheological component of peripheral resistance and contributes to obesity-associated changes in arterial blood pressure [30]. In our study, obesity was associated with a higher risk of abnormal IAD, which is similar to the results of the Kimura A study [10]. Hyperlipidemia was also a risk factor for IAD, but other lipid profiles were not. The multivariate unconditional logistic regression analysis of the hypertensive population showed that their risk factors for abnormal IAD were consistent with those of the general population. Interestingly, smoking and dyslipidemia did not enter into the regression equation, which may be due to the long-term interaction of genetic and environmental factors with hypertension.

In addition, we observed that the risk factors for abnormal IAD were different among the three ethnicities, which may explain the difference in IAD incidence among these ethnicities. In the Han participants, age greater than 45 years, obesity, ABI and diabetes were risk factors. In Uygur participants, smoking was the predominant risk factor, and in the Kazakh participants, obesity and PAD were risk factors. These differences might due to lifestyle, life conditions and genetic factors. A further study is required to better define the risk factors responsible for a higher prevalence of abnormal IAD. Early detection of abnormal IAD may be useful for preventing the progression of atherosclerosis and reducing cardiovascular mortality in Xinjiang, China.

Some limitations of the current study were as follows: First, this was a retrospective, cross-sectional study. Second, the information about diet patterns, physical activity, and socioeconomic conditions were not analyzed in the study population. This could lead to deviations in the results. Systematic, large-scale, prospective studies need to be conducted to further elucidate the epidemiological pattern of abnormal IAD and its associated risk factors.

### Conclusion

There were differences according to ethnicity in the prevalence of IAD in Xinjiang, China. Of the three groups, the Kazakh participants had the highest IAD prevalence and the Han participants had the lowest. Age, obesity and high triglyceride levels were relevant risk factors for IAD. Participants with different ethnicity backgrounds had different patterns of risk factors for IAD.

## Supporting information

S1 Appendix Survey questionnaire. (DOC)

S2 Appendix STROBE checklist. (DOCX)

S1 Table Supplementary table. (DOC)
