# Correction: Epidemiological investigation into the prevalence of abnormal inter-arm blood pressure differences among different ethnicities in Xinjiang, China

**DOI:** 10.1371/journal.pone.0193016

**Published:** 2018-02-12

**Authors:** Ling Sun, Ting Zou, Bao-Zhu Wang, Fen Liu, Qing-Hua Yuan, Yi-Tong Ma, Xiang Ma

The Funding information is incorrect. The correct funding information is as follows: This work was supported by a grant from the National Nature Fund Project (Screening of molecular markers for early diagnosis of acute aortic dissection based on proteomic techniques) awarded to XM (grant number 81660085).
