# Supplementary material for: Epidemiological investigation into the prevalence of abnormal inter-arm blood pressure differences among different ethnicities in Xinjiang, China
Source: PLoS One. 2018 Jan 18;13(1):e0188546. doi: 10.1371/journal.pone.0188546 (PMC5773008; doi:10.1371/journal.pone.0188546)
Supplement: S1 Appendix — (DOC) [file pone.0188546.s001.doc]

**ID Number □□□□□□**

**Life and Health Questionnaire**

**一．Eneral Conditions**

1. Name:

2. Sex: 1=male 2= female □

3. How old are you this year (Zhou Sui)? □□

4. Your date of birth is (year ,month)： □□□□/□□

5. Identity card number: □□□□□□□□□□□□□□□□□□

6. Ethnic groups: 1=Han, 2=Uygur, 3= Kazak, 4=,Hui nationality,5= Mongolian ,

6= other □

7. Occupation: 1= sitting work ,2= standing work activities,3= activity homework

4= farmer, 5= other. □

8. Marital status: 1=unmarried, 2=married or cohabiting, 3= separated or divorced

4= widow or widower,5= other □

The highest educational level: 1= primary school, 2= junior high school

3= high school,secondary school and technical school

4= Junior College，5 = University，6= graduate and above ，9= unknown.

Address:

Zip code: □□□□□□

Contact number: fixed telephone □□□□－□□□□□□□

Mobile phone □□□□□□□□□□□

**二 Personal history**

Do you smoke (now)? 1= do not smoke 2=occasionally 3= regular suction □

You smoke :□ year, □branch/ day:

3. If you have ever smoked, quit smoking and quit smoking now： □year

4.Do you have any passive smoking? 1 =Yes 2 =NO □

5.If you do not drink alcohol (now) 1=No or even 2= often drink drink □

6. How long do you drink? □year

You drink alcohol: □year,□gram/ day,wine (beer, white wine or red wine)?

7.If you have been drank, quit drinking and quit drinking now: □year

8. Do you often drink coffee, tea, cola and so on?

1= never drinks ,2=occasionally drinks, 3=often □

1. Do you have stress? 1 = NO 2 = Yes □
2. Are you menopausal? 1= NO 2=Yes □

11.Such as menopause, menopause years □□

**三 Family history**

1= Have 2=NO have 3=unknown

|  | Father mother Compatriot |
| --- | --- |
| Coronary heart disease | □ □ □ |
| Hypertension | □ □ □ |
| Hyperlipidemia | □ □ □ |
| Diabetes mellitus | □ □ □ |
| Obesity | □ □ □ |
| Stroke | □ □ □ |

1. **Past history (see table below)**

| **A Coronary artery disease** |
| --- |
| 1. Whether Suffering from retrosternal pain 1.No 2.Yes □ |
| 2. Whether have history of myocardial infarction 1.No 2.Yes □ |
| 3. Whether have history of stenting 1.No 2.Yes □ |
| 4. Whether have history of coronary artery bypass grafting 1.No 2.Yes □ |
| 1. Whether still insist on taking the treatment of coronary heart disease drugs   1.No 2.Uncomfortable, take 3.Insist on taking medicine □ |
|  |
| **B Hypertension** |
| 1. Hypertension 1.N0 ( to Dpart ) 2.Yes (to the next questions ) 3.do not know □ |
| 2. Hypertension diagnosis time □□□□years□months |
| 3. Highest blood pressure value □□□/ □□□mmHg |
| 4. Normal blood pressure level □□□/ □□□mmHg |
| 5 .Whether insist on taking antihypertensive drugs in every day  1.No 2.Uncomfortable, take 3.Insist on taking medicine □ |
| 6. Whether have snoring at night or apnea during sleep? 1.N0 2.Yes □ |
| **C Peripheral vascular disease** |
| 1. Whether have limbs that are cold or intermittent claudication 1.N0 2.Yes □ |
| **D Hyperlipidemia** |
| 1. Whether Suffering from hyperlipidemia 1.N0 (transfer your D part) 2.Yes □ |
| 2. Hyperlipidemia diagnosis time □□□□years□months |
| 1. Hyperlipidemia type   1.High TC 2.TG 3.High LDL 4.Low HDL 9. Unknown □+□+□+□ |
| 4. Whether the treatment 1.No 2.Yes 9.unknown □ |
| Drug name: |
| **E Diabetes** |
| Whether suffering from diabetes 1.No ( E to fill in part) 2.Yes □ |
| 2. Diabetes diagnosis time □□□□years□months |
| 3. Types of diabetes 1.1 type diabetes mellitus 2.2 type diabetes mellitus □ |
| 4. Blood glucose control method 1.control diet 2. Insulin 3. hypoglycemic drugs  4. Untreated 9. Unknown □ |
| 5. Normal blood glucose level □□.□mmol/L |
| **F Stroke** |
| 1. Whether suffering from stroke 1.N0 (transfer your three part) 2.Yes □ |
| 2. Stroke diagnosis time □□□□years□months |
| 9. Stroke type 1.cerebral hemorrhage 2.cerebral infarction 9.Unknown □ |
| **G Gout** |
| 1. Whether suffering from gout 1.No (to fill in part H) 2.Yes 9.unknown □ |
| 2. Has been there any swelling or pain in the joints of the toes 1.N0 2.Yes □ |
| 3. Do you like seafood, offal,beeretc and etc 1.N0 2.Yes □ |
| **H History of periodontitis** |
| 1. Whether have a history of periodontitis 1.No 2.Yes □ |
| 2. Whether have brush bleeding or gum spontaneous bleeding 1.No 2.Yes □ |
| 3. Whether have loose teeth 1.No 2.Yes □ |
| **I Kidney history** |
| 1. Whether have ever been diagnosed with kidney disease 1.No 2.Yes □ |
| **J History of taking medicine**  1. Diuretics. 1.N0 2.Yes □ |
| 2. Beta receptor blockers 1.N0 2.Yes □ |
| 3. ACEI 1.N0 2.Yes □ |
| 4. ARB 1.N0 2.Yes □ |
| 5. Calcium antagonists 1.N0 2.Yes □ |
| 6. Alpha receptor blocker 1.No 2.Yes □ |
| 7. Central antihypertensive drug 1.No 2.Yes □ |
| 8. Compound 1.No 2.Yes □ |
| 9. Statins 1.No 2.Yes □ |
| 10. Fibrates 1.No 2.Yes □ |
| 11. Bile acid chelating agent 1.No 2.Yes □ |
| 12. Nicotinic acid and its derivatives 1.No 2.Yes □ |
| 13. Other 1.No 2.Yes □ |

**五．Physical examination**

| 1. Height / Weight □□□ cm/ □□□ kg |
| --- |
| 2. BP □□□/□□□mmHg |
| 3. Waist / hip □□□/□□□cm |
| 4. Electrocardiogram |
| 5. ABI measuremengt ABI(Left: Right: ) PWV(Left: Right: ) |
| 1. Echocardiographic   left ventricular end diastolic diameter: mm; EF: %; ventricular septal thickness:  After the wall thickness of fifteen: mm  carotid intima-media thickness:mm plaques： 1 = □ 2 =□ |
| **7. Exercise ECG** |
| **8.Biochemical tests** |
| Fasting blood glucose： □□.□□ |
| Triglyceride □□.□□mmol/L |
| Total cholesterol □□.□□ |
| LDL □□.□□ |
| HDL □□.□□ |
| Uric acid □□.□□ |
| Urea nitrogen □□.□□ |
| Creatinine □□.□□ |

**Investigator signature:**

**The survey date : □□□□/□□/□□**
