# Supplementary material for: Epidemiological investigation into the prevalence of abnormal inter-arm blood pressure differences among different ethnicities in Xinjiang, China
Source: PLoS One. 2018 Jan 18;13(1):e0188546. doi: 10.1371/journal.pone.0188546 (PMC5773008; doi:10.1371/journal.pone.0188546)
Supplement: S2 Appendix — (DOCX) [file pone.0188546.s002.docx]

STROBE Statement—checklist of items that should be included in reports of observational studies

|  | Item No. | Recommendation | Page  No. | Relevant text from manuscript |
| --- | --- | --- | --- | --- |
| **Title and abstract** | 1 | (*a*) Indicate the study’s design with a commonly used term in the title or the abstract | Page 3 to 10, | By the epidemiological investigation of cardiovascular disease, to investigate the interarm blood pressure difference (IAD) abnormality and its related risk factors among Han, Uygur and Kazakh nationalities in Xinjiang. |
|  |  | (*b*) Provide in the abstract an informative and balanced summary of what was done and what was found | Page 3 to 10, | The incidence of IAD abnormality in Xinjiang is higher than that in other regions of China,there are differences between different ethnic groups elevant risk factors of IAD. |
| Introduction | | | |  |
| Background/rationale | 2 | Explain the scientific background and rationale for the investigation being reported | Page 5, | IAD as a predictor of atherosclerosis is being focused, and its clinical value has been widely recognized in foreign countries,but not in China, due to the lack of large samples and multi-ethnic studies. Therefore,it is important to study the level of IAD in different nationalities and evaluate its related risk factors to prevent cardiovascular diseases. |
| Objectives | 3 | State specific objectives, including any prespecified hypotheses | Page 5, | the purpose of this study was to analyze the abnormal incidence and risk factors of IAD among the Han, Uyghur, and Kazakh in Xinjiang. |
| Methods | | |  | |
| Study design | 4 | Present key elements of study design early in the paper | 窗体顶端  窗体底端  Page 6, | By the epidemiological investigation of cardiovascular disease,to measure IAD and compare the incidence of IAD between different ethnic groups. |
| Setting | 5 | Describe the setting, locations, and relevant dates, including periods of recruitment, exposure, follow-up, and data collection | Page 6, | The Cardiovascular Risk Survey(CRS) study was a multi-ethnic, cross-sectional study designed to investigate the prevalence of risk factors for CVD, and to determine their contribution to the incidence of IAD abnormalityand its related risk factors in the Chinese Han, Uygur, and Kazakh populations in Xinjiang (western China).The selections made from sampling units were based on geographic area, sex, and age groups using household registries. Briefly, the CRS study used a 4-stage stratified sampling method to select a representative sample of the general population in Xinjiang. The research sites included seven cities (Urumqi, Kelamayi, Hetian, Zhaosu, Fukang, Tulufan, and Fuhai). The time period was from October 2007 to March 2010.A total of 11,293 cases with well-recorded IAD were selected, including 5145 Han, 2459 Uyghur, and 3638 Kazak cases. |
| Participants | 6 | (*a*) *Cohort study*—Give the eligibility criteria, and the sources and methods of selection of participants. Describe methods of follow-up  *Case-control study*—Give the eligibility criteria, and the sources and methods of case ascertainment and control selection. Give the rationale for the choice of cases and controls  *Cross-sectional study*—Give the eligibility criteria, and the sources and methods of selection of participants | Page 6, | cross-sectional study designed－all the participants were selected from the Cardiovascular Risk Survey (CRS) study,Briefly, the CRS study used a 4-stage stratified sampling method to select a representative sample of the general population in Xinjiang, northwest of China. The research sites included Urumqi City, Kelamayi City, Fukang City, Turpan Prefecture, Hetian Prefecture, Yili Prefecture. The time period was from October 2007 to March 2010.A total of 11,293 cases with well-recorded IAD were selected, including 5145 Han, 2459 Uyghur, and 3638 Kazak cases. The selections made from sampling units were based on geographic area, sex, and age groups using household registries. |
|  |  | (*b*) *Cohort study*—For matched studies, give matching criteria and number of exposed and unexposed  *Case-control study*—For matched studies, give matching criteria and the number of controls per case |  |  |
| Variables | 7 | Clearly define all outcomes, exposures, predictors, potential confounders, and effect modifiers. Give diagnostic criteria, if applicable | Page6 to9, | 1)Interarm blood pressure difference －IAD was defined as the absolute difference in averaged BPs between the left and right arm, and an IAD ≥ 10 mm Hg was considered to be significant.  2) Hypertension－systolic blood pressure ≥ 140mmHg,(1mmHg =0.133kPa) and (or) diastolic blood pressure ≥ 90 mmHg (according to the criteria in China hypertension guidelines)  3) Obesity－Body mass index = body weight(kg) / height2 (m^2^). Patients with 18.5kg/m^2^ ≤ BMI < 24kg/m^2^ were considered normal weight, patients with 24 kg/m^2^ ≤ BMI < 28 kg/m^2^ overweight, and patients with BMI ≥ 28 kg/m^2^ obese. |
| Data sources/ measurement | 8* | For each variable of interest, give sources of data and details of methods of assessment (measurement). Describe comparability of assessment methods if there is more than one group | Page 6 to 8, | 1）Interarm blood pressure difference －blood pressure of the limbs was measured in a synchronized ay by a Japan's Omron-Colin noninvasive arteriosclerosis analyzer VP-1000 (BP-20w3RPE II), and the IAD was calculated by the blood pressure value of the right upper limb minus that of the left upper limb.  2）Blood pressure measurement－subjects stopped smoking 15 min before the test, with at least 5-min rest in the sitting position. The pressure measurement was performed in the right arm for three times, taking the average of three readings as a mean blood pressure value for statistical analysis.  3）Weight measurement－correction of zero was done before each use. Subjects were fasted and urinated before the measurement. |
| Bias | 9 | Describe any efforts to address potential sources of bias | Page 7, | The data were verified and corrected by two staff members using EpiData3.02 software (EpiData Association, Odense, Denmark). |
| Study size | 10 | Explain how the study size was arrived at | Page 5, | Large epidemiological investigation |

Continued on next page

| Quantitative variables | 11 | Explain how quantitative variables were handled in the analyses. If applicable, describe which groupings were chosen and why | Page 8 to 9, |  |
| --- | --- | --- | --- | --- |
| Statistical methods | 12 | (a) Describe all statistical methods, including those used to control for confounding | Page 9 to 10, | Continuous variables were expressed as mean±standard deviation, numerical data were expressed as rates, and a chi-square test (χ2) was used to evaluate differences between groups.The risk factors for IAD were analyzed using a multivariate unconditional logistic regression |
|  |  | (b) Describe any methods used to examine subgroups and interactions | Page9 to 10, |  |
|  |  | (c) Explain how missing data were addressed |  |  |
|  |  | (d) Cohort study—If applicable, explain how loss to follow-up was addressed  Case-control study—If applicable, explain how matching of cases and controls was addressed  Cross-sectional study—If applicable, describe analytical methods taking account of sampling strategy | Page 9 to 10, |  |
|  |  | (e) Describe any sensitivity analyses |  |  |
| Results | | | | |
| Participants | 13* | (a) Report numbers of individuals at each stage of study—eg numbers potentially eligible, examined for eligibility, confirmed eligible, included in the study, completing follow-up, and analysed | Page 10, |  |
|  |  | (b) Give reasons for non-participation at each stage |  |  |
|  |  | (c) Consider use of a flow diagram |  |  |
| Descriptive data | 14* | (a) Give characteristics of study participants (eg demographic, clinical, social) and information on exposures and potential confounders | Page 14 to 20, | They are from different regions and different populations in  Xinjiang and had a high incidence of risk factors associated with risk factors.Confounding factors may be environmental factors. |
|  |  | (b) Indicate number of participants with missing data for each variable of interest |  | Nobody. |
|  |  | (c) Cohort study—Summarise follow-up time (eg, average and total amount) |  |  |
| Outcome data | 15* | Cohort study—Report numbers of outcome events or summary measures over time |  |  |
|  |  | Case-control study—Report numbers in each exposure category, or summary measures of exposure |  |  |
|  |  | Cross-sectional study—Report numbers of outcome events or summary measures | Page 14 to 20, | he incidence of IAD abnormality in Xinjiang is higher than that in other regions of China, and it increases with age with the influence of ethnic differences.  IAD incidence in the Kazak population is higher than that in the Uygur and the Han populations.Increased age, TGs, and obesity appear to be relevant risk factors of IAD. |
| Main results | 16 | (a) Give unadjusted estimates and, if applicable, confounder-adjusted estimates and their precision (eg, 95% confidence interval). Make clear which confounders were adjusted for and why they were included | Page 14 to 20. | After adjusting the factors such as investigation site, sex, marital status, and diet,compared with the age group of 35-44 years (OR=1), the risk of developing IAD increased by 1.71-fold in the age group of over 75 years (OR=1.71, 95%CI: 1.25-2.33).  In the obese group, the risk of IAD increased 1.41-fold (OR=1.41, 95%CI: 1.21-1.65) compared with the normal population.The reason for the inclusion is that they are a significant risk factor for IAD. |
|  |  | (b) Report category boundaries when continuous variables were categorized |  | For patients in the age groups of 35-44, 45-54, 55-64, 65-74, and over 75 years, |
|  |  | (c) If relevant, consider translating estimates of relative risk into absolute risk for a meaningful time period |  |  |

Continued on next page

| Other analyses | 17 | Report other analyses done—eg analyses of subgroups and interactions, and sensitivity analyses |  |  |
| --- | --- | --- | --- | --- |
| Discussion | | | | |
| Key results | 18 | Summarise key results with reference to study bjoectives | Page 21 | This study aimed to investigate the interarm blood pressure difference (IAD) abnormality and its related risk factors among Han, Uygur and Kazakh nationalities in Xinjiang by measuring IAD. |
| Limitations | 19 | Discuss limitations of the study, taking into account sources of potential bias or imprecision. Discuss both direction and magnitude of any potential bias | Page 25, | Some limitations of the current study were as follows: First, this was a retrospective cross-sectional study. Second, the information about diet patterns, physical activity, and socioeconomic condition was not analyzed in the study population. |
| Interpretation | 20 | Give a cautious overall interpretation of results considering objectives, limitations, multiplicity of analyses, results from similar studies, and other relevant evidence | Page 21to 25 | The results of this study show that The incidence of IAD abnormality in Xinjiang is higher than that in other regions of China, and it increases with age with the influence of ethnic differences.The incidences of IAD abnormalities among the three ethnic groups were statistically different .IAD incidence in the Kazak population is higher than that in the Uygur and the Han populations.the risk factors for IAD were increased age,obesity triglyceride, and ankle-brachial index.Increased age, TGs, and obesity appear to be relevant risk factors of IAD.  Previous studies have shown that as age increases, arterial elasticity decreases and peripheral vascular resistance increases, leading to atherosclerosis and increased incidence of IAD in the elderly population . This is consistent with the results of the present study. Therefore, the study suggests that people over the age of 45 are at high risk of IAD, and should pay attention to the prevention of IAD. |
| Generalisability | 21 | Discuss the generalisability (external validity) of the study results | Page 25. |  |
| Other information | |  | | |
| Funding | 22 | Give the source of funding and the role of the funders for the present study and, if applicable, for the original study on which the present article is based | - | No |

*Give information separately for cases and controls in case-control studies and, if applicable, for exposed and unexposed groups in cohort and cross-sectional studies.

**Note:** An Explanation and Elaboration article discusses each checklist item and gives methodological background and published examples of transparent reporting. The STROBE checklist is best used in conjunction with this article (freely available on the Web sites of PLoS Medicine at http://www.plosmedicine.org/, Annals of Internal Medicine at http://www.annals.org/, and Epidemiology at http://www.epidem.com/). Information on the STROBE Initiative is available at www.strobe-statement.org.
