# Supplementary material for: Epidemiological investigation into the prevalence of abnormal inter-arm blood pressure differences among different ethnicities in Xinjiang, China
Source: PLoS One. 2018 Jan 18;13(1):e0188546. doi: 10.1371/journal.pone.0188546 (PMC5773008; doi:10.1371/journal.pone.0188546)
Supplement: S1 Table — (DOC) [file pone.0188546.s003.doc]

**Supplementary Table 1. Multivariate unconditional logistic regression analysis for risk factors of IAD in the three ethnicities.**

|  |  | Han |  |  |  | Uygur |  |  |  | Kazakh |  |
| --- | --- | --- | --- | --- | --- | --- | --- | --- | --- | --- | --- |
|  | OR | 95%CI | P |  | OR | 95%CI | P |  | OR | 95%CI | P |
| Sex | 1.027 | 0.81-1.31 | 0.83 |  | 0.88 | 0.64-1.21 | 0.44 |  | 0.99 | 0.79-1.25 | 0.94 |
| Age |  |  | 0.01 |  |  |  | 0.58 |  |  |  | 0.36 |
| 35-44y | 1 |  |  |  | 1 |  |  |  | 1 |  |  |
| 45-54y | 1.29 | 1.01-1.66 | 0.04 |  | 1.20 | 0.86-1.69 | 0.28 |  | 1.20 | 0.94-1.55 | 0.15 |
| 55-64y | 1.41 | 1.08-1.85 | 0.01 |  | 1.27 | 0.88-1.84 | 0.20 |  | 1.24 | 0.94-1.65 | 0.14 |
| 65-74y | 1.64 | 1.24-2.16 | 0.00 |  | 1.40 | 0.91-2.15 | 0.12 |  | 1.22 | 0.87-1.72 | 0.26 |
| More than 75y | 1.70 | 1.11-2.60 | 0.02 |  | 1.40 | 0.71-2.78 | 0.33 |  | 1.62 | 0.91-2.89 | 0.10 |
| Smoking | 1.00 | 0.77-1.29 | 0.99 |  | 1.83 | 1.20-2.79 | 0.01 |  | 0.96 | 0.76-1.20 | 0.71 |
| Drinking | 1.08 | 0.83-1.40 | 0.59 |  | 1.47 | 0.95-2.29 | 0.08 |  | 0.87 | 0.64-1.18 | 0.36 |
| BMI |  |  | 0.01 |  |  |  | 0.47 |  |  |  | 0.007 |
| Normol | 1 |  |  |  | 1 |  |  |  | 1 |  |  |
| Overweight | 1.16 | 0.94-1.44 | 0.17 |  | 1.12 | 0.82-1.54 | 0.46 |  | 1.18 | 0.91-1.52 | 0.22 |
| Obsesity | 1.47 | 1.15-1.88 | 0.002 |  | 1.23 | 0.89-1.70 | 0.22 |  | 1.49 | 1.15-1.92 | 0.002 |
| Diabetes | 1.34 | 1.01-1.78 | 0.045 |  | 1.00 | 0.60-1.67 | 0.70 |  | 0.88 | 0.19-2.02 | 0.62 |
| PAD | 0.93 | 0.56-1.57 | 0.79 |  | 1.18 | 0.72-1.94 | 0.51 |  | 1.60 | 1.01-2.53 | 0.04 |
| ABI | 0.23 | 0.08-0.70 | 0.009 |  | 0.84 | 0.20-3.52 | 0.81 |  | 0.61 | 0.19-2.02 | 0.42 |
| TG | 1.14 | 0.94-1.39 | 0.19 |  | 1.24 | 0.95-1.62 | 0.11 |  | 1.08 | 0.83-1.40 | 0.57 |
| TC | 0.92 | 0.76-1.12 | 0.42 |  | 0.99 | 0.80-1.35 | 0.53 |  | 0.97 | 0.79-1.20 | 0.80 |
| LDL-c | 1.05 | 0.87-1.26 | 0.17 |  | 0.90 | 0.69-1.17 | 0.43 |  | 0.95 | 0.78-1.16 | 0.63 |
| HDL-c | 0.97 | 0.80-1.18 | 0.77 |  | 1.04 | 0.80-1.35 | 0.77 |  | 1.08 | 0.88-1.34 | 0.46 |

PAD, Peripheral arterial disease; ABI, Ankle-brachial index; BMI, body mass index; HDL-c, high-density lipoprotein-cholesterol; LDL-c, low-density lipoprotein-cholesterol; TC, total cholesterol; TG, triglycerides.
